# Anemia and its associated factors among adult people living with human immunodeficiency virus at Wolaita Sodo University teaching referral hospital

**DOI:** 10.1371/journal.pone.0221853

**Published:** 2019-10-09

**Authors:** Temesgen Anjulo Ageru, Mengistu Meskele Koyra, Kassa Daka Gidebo, Temesgen Lera Abiso

**Affiliations:** 1 Wolaita Sodo University Teaching Referral Hospital, Wolaita Sodo, South Ethiopia; 2 School of Public Health, College of Medicine and Health Sciences, Wolaita Sodo University, Wolaita Sodo, South Ethiopia; University of Texas Health Science Center at San Antonio, UNITED STATES

## Abstract

**Background:**

In Sub-Saharan Africa, both HIV/AIDS and anemia have considerable public health problems. Anemia has an adverse effect on treatment outcome and it decreases the quality of life among adult HIV patients. This study was aimed to assess the prevalence of anemia and its associated factors among adult HIV positive patients in Wolaita Sodo University Teaching Referral Hospital.

**Method:**

Institution based cross-sectional study was conducted at Wolaita Sodo University Teaching Referral Hospital from 01 October to December 30, 2016. A randomly selected 411 adult people living with the human immunodeficiency virus were included in the study. A pre-tested questionnaire was used to collect data. Variables with *P-value* ≤0.25 in the bivariable logistic regression model were taken into multivariable logistic regression analysis along with 95% confidence interval and Odds Ratio was used to examine the association between anemia and independent variables. *P*˗value ≤ 0.05 was taken as statistically significant.

**Result:**

Prevalence of anemia in this study was 36.5% with 95% CI (32%-41%). Factors associated with anemia among adult people living with HIV/AIDS were individuals who lived with HIV ≥9years (AOR = 2.6, 95% CI:-1.03–6.59),years lived with HIV 5–8 years (AOR = 2.59, 95% CI:-1.02–6.57),CD4 count <200cells/ul (AOR = 4.2, 95%CI:-2.03–8.67), CD4 count200-350cells/ul(AOR = 1.82,95%CI:-1.01–3.26),infection with intestinal parasites (AOR = 2.04, 95% CI:-1.06–3.95), Participants with BMI <18.5kg/m^2^ (AOR = 2.96, 95%CI:-1.37–6.390),BMI 18.5-25kg/m^2^(AOR = 1.98, 95%CI:-1.11–3.56) and being HAART naïve (AOR = 2.23, 95% CI:- 1.16–4.28).

**Conclusion:**

Prevalence of anemia among this study participant was high. This may affect the treatment outcome, increases morbidity and mortality of the participants. So periodic screening of anemia, a routine checkup of nutritional status, CD4 count and examination for intestinal parasite are essential.

## Introduction

World Health Organization(WHO) define anemia as hemoglobin (Hgb) less 12g/dl for women and 13g/dl for men[1]. It is a condition in which the number of red blood cell decreased to meet the body's physiological needs. This leads to an insufficient oxygen-carrying capacity of red blood cells[2].

Anemia is a common hematological complication seen in people with HIV/AIDS infection and it can have serious implications, which may vary from functional and quality of life decrements to an association with disease progression and decreased survival[3,4].

The mechanism of HIV infection is cytotoxic to T -helper cell lymphocytes that lead dysfunction of B Cells and altered release of cytokines. HIV infected T cells directly suppress the bone marrow progenitors which suppressing hemopoiesis, thus leads to anemia[5]. HIV can destroy CD4+(cluster of differentiation) T cells directly as well as indirectly but may lie relatively dormant in macrophages for long periods with intermittent reactivation[6].

All HIV positive patients regardless of WHO clinical staging and CD4 count, initiated the treatment after baseline CD4 counts and they start first line antiretroviral drugs TDF + 3TC (or FTC) + EFV and TDF + 3TC (or FTC) + NVP, AZT + 3TC + EFV, AZT + 3TC + NVP[7].

Anemia is a public health problem affecting both developed and developing countries with major consequences on human health as well as economic and social growth. Globally about 2 billion people are estimated to be suffering from anemia. It is responsible for 1 million deaths per year in Sub Saharan Africa and South East Asia[8,9].

In sub-Saharan Africa, where the prevalence of HIV/AIDS and malnutrition are high, anemia is common adding to the considerable burden of morbidity and mortality. The problem of anemia in many resource-poor settings is made worse by the fact that population characteristics such as density, growth and socio-economic factors. In addition, the infections like malaria and intestinal parasites add to the burden of anemia[10,11].

The prevalence of anemia varies in HIV positive patients with clinical AIDS range 63–95% in different studies[12,13]. As HIV disease progresses, the prevalence and severity of anemia also increase as studies in different countries and within countries accordingly[14].

Ethiopia is one of the HIV/AIDS-affected countries in sub- Saharan Africa, with more than 1.3 million people living with HIV. In Ethiopia, the adult prevalence of HIV was estimated to be 1.5% in 2011[15,16]. Some studies in Ethiopia indicates that 38% of men and 62% of women living with HIV/AIDS were anemic[17,18].

Several factors play a role in the development of anemia in patients with HIV, including chronic disease, opportunistic infections, nutritional deficiencies and toxicities from medications. One of the factors related is the adverse side effect of antiretroviral therapy. Since its start, ART brought many benefits but it leaves this challenge[14,19]. There are drugs that worsen anemia in HIV/AIDS patients including Zidovudine (AZT) and Stavudine (d4T)[20,21].

Anemia in HIV infection can be the product of a number of interactive factors and it has been difficult to determine the sequence or order of their impact in terms of causality. It is possible that HIV/AIDS is a major causative factor in the development of anemia and anemia is indicative of the likely severity or progression of the disorder[22].

Studies conducted in Ethiopia on the prevalence of anemia among HIV positive patients in different areas showed different prevalence: in Western 23.1%, Gondar hospital on HAART 11.7% and HAART naïve 29.7%, Addis Ababa on HAART patients was 33%[13,23,24].

The study done in Ethiopia, Zewuditu Memorial Hospital showed that factors associated with anemia before initiation of HAART were male sex, age greater than 55,WHO Clinical stage III/IV,BMI <18.5kg/m2 and the presence of Opportunistic infections such as TB and candidiasis, Lower CD4 count was also associated with anemia. Whereas factors associated with anemia after initiation of HAART were being male sex, drug regimen, increased age and CD4 count less than 200cell/ul and 200-350cell/ul[17,25].

While there is a wide variation in the prevalence of anemia and its associated factors among HIV/AIDS positive patients in different studies in the countries, there is an insufficiency of information on the prevalence and associated factors of anemia among HIV/AIDS adult patients in Ethiopia. Adult people living with HIV are not given attention in the world as well as in Ethiopia. Due to this reason, there is high morbidity and mortality among adults which needs attention. The finding of this study is important for policymakers, organizations working on prevention of HIV/AIDS-related deaths, ART clinic managers and service providers to improve the health status of adults living with HIV. Therefore, this study aimed to assess the prevalence of anemia and its associated factors among adult People living with Human immunodeficiency virus attending an ART clinic at Wolaita Sodo University Teaching Referral hospital, South Ethiopia.

## Method and materials

### Study setting

The study was conducted at Wolaita Sodo University Teaching Referral Hospital (WSUTRH), which is located in Wolaita Zone, 154km from Hawassa, capital of South nations, nationalities and peoples region, and 329km from Addis Ababa, capital of Ethiopia. The hospital provides general outpatient and inpatient services including medical, surgical, pediatric, psychiatric, Ophthalmic, Emergency, Gynecology and Obstetrics care. It also provides testing and treatment of HIV/AIDS including voluntary counseling and testing, provider-initiated counseling and testing, prevention of HIV transmission from mothers to children and ART.

The ART program of the hospital was launched in 2005. When patients diagnosed as HIV positive in one of the above counseling and testing protocols, they are linked to the ART clinic using an internal facility referral form. Then patients are registered in ART logbook according to disease progression for HAART naive and ART follow up. When patients initiated HAART, they are routinely followed every two weeks in the beginning, then every month and then every three months for HAART dispensing and clinical checkup.

Currently, there are 2000 HIV patients under the ART service of which 1500 patients are on HAART and about 500 patients on HAART naive care.

### Study design and period

An institution-based cross-sectional study was conducted from 01 October to 30 December 2016.

### Population

All adult people living with HIV aged 15 years old and above attending ART clinic in Wolaita Sodo University Teaching Referral Hospital, South Ethiopia.

### Study population

Randomly selected HIV positive adults attending the ART clinic at WSUTRH who fulfill inclusion criteria during the study period.

### Eligibility

Adults living with HIV who are 15 years old and above attending ART clinic to their routine follow up during the study period were included and patients who are critically ill and pregnant women were excluded from the study.

### Sample size calculation

The sample size was calculated using single population proportion formula by open Epienfo303 Software with the assumption of confidence level = 95% (α = 0.05) z = 1.96, Margin of error (desired level of absolute precision) d = 5%. Non-response rate-10%. Prevalence of anemia among HIV positive adult patients 41**.2%,** in eastern Ethiopia [26].Calculated sample size was 373 and adding 10% of non-response rate = 373+38 = 411. Therefore, the maximum sample size taken was 411.

### Sampling procedures

Simple random sampling technique was used to select study participants from the ART Clinic database. All adults listed in the database, then each individual was assigned using Medical Record Number (MRN) and the assigned number sorted. Individual MRN was randomly selected. Randomly selected individual by MRN was listed, printed out and first n chosen by lottery method. The repeated individual was rejected and controlled by MRN.

### Data collection technique

After getting informed written consent from each study participants, pre-tested structured questionnaire was used to collect data on the socio-demographic characteristics, clinical factors, and anthropometric measurements. Laboratory technologists collected 4 ml of whole blood using EDTA anti-coagulated tests tube from each study participants. Then 1 gram of stool specimens collected from each participant. Complete blood cell and CD4+ cell counts were done using BC-3000 Plus hematological analyzer and BD FACS count machine respectively. Thick blood film and stool examination were performed for malaria and intestinal parasites respectively.

We used the WHO Stepwise Approach to Chronic Disease Risk Factor Surveillance for anthropometric measurements[27]. Measurement of weight was conducted using a standard scale that is applicable for weight measurement in medical setups.

The scale pointer was checked at zero before taking every measurement. The person was required to dress in light clothes. Women were asked to remove a scarf. He/she stood straight and unassisted on the center of a balance platform. The average measurement of weight was recorded to the nearest 0.1 kg.

Height was measured using a standard scale. The study participants were asked to remove their shoes, stood erect, the position at the plane with feet together and knee straight. The heels, buttock, shoulder blades and the back of the head were in touch against the vertical stand of the stadiometer and the values were recorded to the nearest 0.1cm. Body mass index (BMI) was calculated using weight and height (Kg/m^2^).

### Operational definition

**Anemia:** is a condition in which hemoglobin level less than 13 g/dl for male and 12g/dl female according to WHO[2].

### Variable of study

Dependent variable is anemia based on Hgb Status and independent variables are age, sex, monthly income, occupation, HAART Status, educational status, residency, clinical stage of disease, years lived with HIV, co-morbidity with TB and candidiasis, intestinal and malaria parasites, CD4 count, drug regimen type, nutritional status, meal intake and dietary intake of patients.

### Data quality control

To assure the quality of data, two days orientation was given for nurses, laboratory Technologists and supervisors on the technique of interviewing and sample collection. Data collection was supervised daily during questionnaire filling and sample collection. Starting from sample collections to all laboratory result delivery, Standard Operating Producers (SOP) was followed. The expiry date of all reagents and chemicals were rechecked. Daily quality control low, medium and high were run for hematology analyzer for haemoglobin count and BD FACS count for CD4 Machines. Based on their SOPs quality of Giemsa stain was checked by doing known malaria positive and negative control blood film. For stool analyses, wet mount, normal saline was checked for expired date and done according to manufactures instruction. Discussion held every day after the data collection between data collectors, supervisors, and the principal investigator about problems faced during the data collection process and the problem resolved with a common memorandum of understanding. Pre-testing was conducted in 5% (20 individuals) of the total sample size in Sodo Health Centre and some corrections were made. Questionnaires were translated from English to Amharic and translated back to English to check for consistency and accuracy.

### Data management and analysis

After the data collection, questionnaires were checked for completeness and consistency on a daily basis. Data was manually edited before data entry, entered into Epi-Info 3.5.4 and finally, the data was exported to SPSS version 20.

Data analysis was done by using SPSS 20.0 version. Descriptive analysis was done using frequency tables. A bivariable analysis was carried out to look for a statistical association between each independent variables and dependent variable. Those variables with a p-value less than 0.25 in the bivariable logistic regression analysis model were taken to multivariable logistic regressions to control confounders. Finally, multivariable analysis was carried out to identify independent predictors of the outcome variable and adjusted odds ratio with 95% CI used. Then, p-value ≤ 0.05 was taken to claim statistical significance. Multi-co linearity among independent variables was checked for a variance inflation factor. The model was tested by Hosmer and Lemeshow goodness of fit tests.

### Ethical consideration

The ethical clearance was obtained from the Ethical review committee of Wolaita Sodo University, college of health science and medicine. Formal permission was also obtained from Wolaita Sodo University Teaching Referral Hospital. Study participants whose age 18 and above were informed about the study purpose and written consent were signed; the age between 15-17yrs clearly informed and assent form signed from both guardians and study participants. They have the full right to participate or not to participate. The participants also were told that they might stop participation during any time from the study during data collection. Withdrawal from the study was not harming their routine HIV care follow-up. In addition to this, the blood or stool samples taken to this study were not used any other study or purpose. The findings kept confidential in a secured locker and the positive results were timely reported to the clinicians for appropriate interventions.

## Result

### Socio-demographic characteristics

A total of 411 HIV positive study participants included in this study, making a response rate of 100%. Two hundred fifty-eight (62.8%) study participants were females and the mean (±SD) age of the study participants was 35.03± 8.9 years and the highest portion of age was 25–44 years, which accounted 308 (74.93%) of total participants. Regarding their residency, 365 (88.8%) was living in an urban setting. Based on their marital status, 274(66.7%) of study the participants were married. Eighty-three (20.1%) of study participants had no education and 256(62.3%) had a monthly income less than 750 ETB S1 Table.

### Clinical characteristics and drug regimens

From the total of 411 HIV positive adult, minimum years lived with HIV was 2 months and maximum of 23 years. According to their WHO stage, 298(96.8%) from HAART user and 94(91.3%) on HAART naive were on stage one, while none of the study participants were on WHO stage four. Total of 411 study participants included in this study, 23 (5.6%) had clinical comorbidity; from comorbidity identified TB, candidiasis, malaria and diarrheal disease were major ones. Of the total, 13.6% participants had a CD4 count less than 200cell/ul.

Among 411 study participants, 103 were on HAART naive and 308 HAART users. From HAART user, 152 (49.4%) had a treatment duration of 60–120 months/5-10years/, 22.7% had a duration of more than 120 months (10years). During the course of the treatment, 106 (34.4%) of HAART user had switched their previous drug regimen and 77 (72.6%) of them were switched D4T/3TC/NVP, 15(14.2%) D4T/3TC/EFV and, 7(6.6%) AZT/3TC/NVP. Based on their treatment type, 165(53.6%) and 81(26.3%) were taking TDF/3TC/EFV, AZT/3TC/NVP respectively. According to the drug regimen, 284(92.2%) were on the first line drug regimen and 24(7.8%) on the second line. One hundred three (25%) were on HAART naive S2 Table.

### Meal frequency and nutritional factors

A total of 411 HIV positive study participants included in this study, 14.6% of study participants had BMI less than 18.5kg/m^2^.

Three hundred eighty-eight (94.4%) of the respondent had nutritional counseling and awareness. One hundred eleven (27.7%) participants had participated in the supplementary food program.

It was found that 4.9% of participants had eaten two or less than 2 per day and 61.3% of never ate snack meal. Regarding food diversity, 283 (68.9%) of ate food group three and less than three S3 Table.

### Food consumption pattern

Majority of the study participants 93.9% of participant ate cereal food type four and more than 4 times per week. More than half (68.4%) of study participants consume roots and tuber in their daily meal. Third-fourth (89.3%) of respondents had not eaten fish even once in the month S4 **Table**.

### Prevalence of intestinal and malaria parasites

Stool and blood film examined for 411 HIV positive respondents included in this study, overall 12.2% (11.7% of HAART users and 13.6% participants on HAART naive were infected with intestinal parasites. Among intestinal parasites identified, *E*.*histolytica* is the most prevalent 55.5% and the least was *G*.*lamblia* 5.5%. Seven (1.6%) of study participants were infected with malaria parasites, four *P*. *vivax*, and three *P*. *falciparum* species.

### Hemoglobin determination based sex and severity of anemia

From 411 HIV positive study participants included in this study, the overall prevalence of anemia was 150 (36.5% with 95% CI:-32%-41%). Based on their sex, 36.6% of male and 36.4% of female were anemic. According to severity, 70.7% of participants had mild anemia and 2.6% had severe. On the other hand, 5.3% of male on HAART naive and 4.5% of female on HAART were severely anemic S5 Table

### Factors associated with anemia among study participants

Candidate variables were sex, marital status, educational status; Years lived with HIV, eating difficulty, HAART status, CD4 count, infection with intestinal parasites and BMI. However, from these variables, Educational status, years lived with a virus, CD4 count, infection with intestinal parasites, HAART status and BMI <18.5% were predictors of anemia.

Study participants lived with HIV ≥9years were 2.6 times more likely to develop anemia than individual lived with HIV <2years (AOR = 2.6, 95%CI:-1.03–6.59) and participants lived with HIV 5–8 years were 2.59 times more likely to develop anemia than individual lived with HIV < 2 years (AOR = 2.59, 95% CI:-1.02–6.57. Having CD4 Count <200cells/ul were 4.2 times more likely to be anemic (AOR = 4.2, 95%CI 2.09–8.67) than individuals with CD4 count >500cells/ul and individuals having CD4 count 200-350cells/ul were 1.82 times more likely to be anemic(AOR = 1.82 95% CI:-1.01–3.26) than individuals with CD4 count > 500cells/ul. Individuals infected with intestinal parasites were 2.04 times more likely to be anemic (AOR = 2.04, 95% CI:-1.06–3.95 than their counterparts were. Participants with BMI <18.5 were 2.96times chance to have anemia (AOR = 2.96, 95% CI:-1.37–6.39) and Participants who were HAART naïve were 2.23 times more likely to develop anemia (AOR = 2.23, 95% CI 1.16–4.28) than those who were on HAART S6 **Table**.

## Discussion

The overall prevalence of anemia among 411 HIV positive adult study participants was 36.5% with 95% CI (32%-41%). It was 34.4% among HAART user and 41.7% on HAART naive study participants. BMI <18.5kg/m2, CD4 count lower than 200cells/ul, years lived with virus and infection with the intestinal parasite were factors associated with anemia among study participants. The finding of this study was higher compared to other studies conducted Ethiopia, in Western Ethiopia overall prevalence of anemia was 23.1% (16.2% on HAART user and 29.9% on HAART naive care)[13] and in Northern Ethiopia, its prevalence was11.7% on HAART and 29.7% in HAART naive in Gondar University Hospital[24]. This could be due to the selection of the comparison group used and age group of study participants.

This finding is lower than the study conducted in Ghana Regional Hospital in Bolgatang, among HAART and HAART naive care was 46% and 63% respectively[28], India, overall prevalence of anemia was 77.5%[29], Middle East, Iran 71.0%[30] and Nigeria Federal Medical center overall prevalence was 64%[31]. This may be due to geographical location, study design, years the study participants lived with the virus and the study period in previous studies.

Having low CD4 count became significantly associated with anemia in this study. A similar association was seen in West Ethiopia, Jima University specialized hospital[32], in North Ethiopia Gondar university hospital[24], South Africa Johannesburg[33]. This is due to lyses and decreased production of RBC resulting in low Hgb with the advancement of HIV related diseases.

Participants with a BMI <18.5 kg/m^2^ were significantly associated with anemia in the current study. Similar reports noticed in Addis Ababa[34], South Africa Johannesburg[33], in Gondar[35], in Tanzania Dar Salem[36], in two West Africa Countries [37].This may be due to deficiencies of many micronutrients, including iron, folate, B12, and vitamin A, which contribute directly to anemia.

Being HAART naïve was found to be associated with anemia in this study. This finding is in agreement with the findings from other studies in Ethiopia[13,38], Ghana[3] and South Africa [33].This can be explained by the effects of the HAART. HAART can suppress HIV, a virus which is known to affect the bone marrow; so by suppressing the viral load, HAART could prevent anemia. On the other hand, HAART could improve the immunity of PLHIV by decreasing the occurrence of multiple OIs, which are identified to potentially cause anemia.

In the present study, participants lived with HIV 5–8 years and ≥ 9 years showed significant association with anemia. This is supported by another study in West Ethiopia[13] and South Africa[22]. This might be due to decreased red blood cell production or increased destruction of Red blood cells, the direct effect of HIV infection itself and myelosuppression of antiretroviral therapy medication.

Infection with intestinal parasites was associated with anemia among study participants and this finding is supported many studies conducted in Ethiopia, West South Ethiopia[13], in northern Ethiopia, Dessie Hospital[39], in South Ethiopia Butajira Hospital[40], in Ahaz health center, south of Iran[41] and in Uganda[42].This might be due to parasites interruption of nutrients absorption to the body and blood loss associated with parasitic infections.

In this study, using Zidovudine (AZT) or d4T (Stavudine) based drug regimen did not show significant associated with anemia, which is different from study conducted in elsewhere, Addis Ababa Zewuditu Hospital [20], in South Africa[33], in Rural Tanzania[43], in eastern India[44], in Phnom Penh, Cambodia [45]. The difference in this study could be due to a small proportion of study participants were on AZT based regimen.

Contrary to previous studies in Ethiopia [40], in Rwanda [46], in Ghana[47] study participants with malaria parasite did not show any association with anemia in the present study. This might be due to a few study participants infected with malaria in this study, which is none comparable with the previous study.

Another factor that did not show any significant association with anemia in this study was co-infection with TB, which is different from a study conducted in South Africa Cape Town [48], in Tanzania Dar Salem [49]. This could be also the small number of study participants co-infected with TB (only 0.7%).

A study conducted in the University of Kerala, India showed 64% of study participants were unaware of nutritional aspects[50]. Dietary diversity and food frequency did not show any association with anemia in this study. This could be due to overall 94.4% study participants, 95.8% of study participants among HAART user and 90.3% of HAART naive care got nutritional counseling during routine HIV care. Another possible reason is assessing dietary pattern in developing countries is difficult due to recall bias and study participants may not have unique feeding practice.

## Limitation of this study

Recall bias in 24hrs dietary intake may present, Vit.B12 deficiency, the major nutritional anemia was not tested because of limited setup and hemoglobin adjustment for altitude was not done.

## Conclusion

Prevalence of anemia among these study participants is high; this affects the treatment outcome, increases morbidity and mortality of the participants. Years lived with HIV ≥9yrs, years lived with HIV 5–8 yrs, CD4 count<200cells/ul, CD4 count 200-350cells/ul, infection with intestinal parasites and participants with BMI <18.5kg/m2, BMI 18.5-25kg/m2 and Being HAART naïve were factors associated with anemia among adult people living with HIV/AIDS in this study.

## Recommendation

ART clinic should conduct periodic screening of anemia, a routine checkup of intestinal parasites examination and routine checkup of CD4among patients with HIV.

Nutritional status of the clients should be checked in each visit and patients on chronic care for a long period would be carefully followed for anemia and comprehensive care and treatment strategy of anemia should be established.

Governmental and Nongovernmental organizations working on prevention of HIV/AIDS-related programs should give attention to adults living with HIV.

Conducting other longitudinal research is recommended

## Supporting information

S1 Table Socio-demographic characteristics of PLHIV at WSUTRH, South Ethiopia, December 2016. *others–students and farmer
(DOCX)

S2 Table Clinical characteristics among PLHIV at WSUTRH, South Ethiopia, December 2016. *TDF/3TC/Atzanavir/kalteza, AZT/3TC/Atzanavir/kalteza and ABC/3TC/ATV/r are second line drugs. Note:—* 0 value in the above table indicates patients who were on HAART naive not yet started drug treatment
(DOCX)

S3 Table Nutritional status and meal frequency among PLHIV at WSUTRH, South Ethiopia, December 2016. BMI-Body Mass Index
(DOCX)

S4 Table Food Consumption Pattern among PLHIV at WSUTRH, South Ethiopia, December (n = 411). (DOCX)

S5 Table The prevalence of anemia based on sex and HAART status at WSUTRH, South Ethiopia, December 2016(n = 411). (DOCX)

S6 Table Factors associated with Anemia among adult people living with HIV at WSUTRH, South Ethiopia, December, 2016. *P value ≤0.05 for AOR, BMI-Body Mass Index, HAART-Highly Active Antiretroviral Therapy, CD4-Cluster of Differentiation at T-cell.
(DOCX)

S1 Study Tool (DOCX)

S1 SPSS Out Put (RTF)

S1 SPSS Descriptives Out Put (DOC)

## Abbreviation and acronyms

ABC: Abacavir
AIDS: Acquired Immuno Deficiency Syndrome
ART: Antiretroviral Therapy
AZT: Zidovudine
BMI: Body Mass Index
CD4: Cluster of Differentiation at T-cell
4DTC: Stavudine
EDHS: Ethiopia Demographic and Health Survey
ETB: Ethiopian Birr
FACS: Fluorescent Activated Cell Sorts
FMOH: Federal Ministry of Health
HAART: Highly Active Antiretroviral Therapy
HGB: Hemoglobin
HIV: Human Immunodeficiency Virus
PLHIV: People Living With Human Immunodeficiency Virus
PMTCT: Prevention of Mother to Child Transmission of HIV/AIDS
RBC: Red Blood Cell; 3TC- Lamivudine
TB: Tuberculosis
TDF: Tenofovir Disofroxil
UN: United Nation
UNAIDS: Joint United Nations Programme on HIV/AIDS
WBC: White Blood Cell
WHO: World Health Organization
WSUTRH: Wolaita Sodo University Teaching Referral Hospital
