# Supplementary material for: Anemia and its associated factors among adult people living with human immunodeficiency virus at Wolaita Sodo University teaching referral hospital
Source: PLoS One. 2019 Oct 9;14(10):e0221853. doi: 10.1371/journal.pone.0221853 (PMC6785157; doi:10.1371/journal.pone.0221853)
Supplement: S1 Table — *others–students and farmer (DOCX) [file pone.0221853.s001.docx]

| ***Variables (n=411)*** | | ***Frequency*** | ***Percent (%)*** |
| --- | --- | --- | --- |
| ***Sex*** | ***Male*** | ***153*** | ***37.2*** |
|  | ***Female*** | ***258*** | ***62.8*** |
| ***Age*** | ***15-24*** | ***39*** | ***9.5*** |
|  | ***25-44*** | ***308*** | ***74.9*** |
|  | ***45+*** | ***64*** | ***15.56*** |
| ***Residency*** | ***Urban*** | ***365*** | ***88.8*** |
|  | ***Rural*** | ***46*** | ***11.2*** |
| ***Marital status*** | ***Married*** | ***274*** | ***66.7*** |
|  | ***Single*** | ***59*** | ***14.4*** |
|  | ***Divorced*** | ***34*** | ***8.3*** |
|  | ***Widow*** | ***44*** | ***10.6*** |
| ***Education status*** | ***No education*** | ***83*** | ***20.21*** |
|  | ***Primary*** | ***130*** | ***31.63*** |
|  | ***Secondary*** | ***139*** | ***33.81*** |
|  | ***Tertiary*** | ***59*** | ***14.35*** |
| ***Occupations*** | ***No occupation*** | ***72*** | ***17.5*** |
|  | ***Employed*** | ***131*** | ***31.9*** |
|  | ***Housewife*** | ***70*** | ***17.0*** |
|  | ***Merchants*** | ***82*** | ***19.9*** |
|  | ***Daily laborer*** | ***28*** | ***6.8*** |
|  | ***Others**** | ***28*** | ***6.8*** |
| ***Monthly income in ETB*** | ***<750*** | ***256*** | ***62.3*** |
|  | ***750-1600*** | ***58*** | ***14.1*** |
|  | ***>1600*** | ***97*** | ***23.6*** |

S1 Table
