# Supplementary material for: Anemia and its associated factors among adult people living with human immunodeficiency virus at Wolaita Sodo University teaching referral hospital
Source: PLoS One. 2019 Oct 9;14(10):e0221853. doi: 10.1371/journal.pone.0221853 (PMC6785157; doi:10.1371/journal.pone.0221853)
Supplement: S2 Table — *TDF/3TC/Atzanavir/kalteza, AZT/3TC/Atzanavir/kalteza and ABC/3TC/ATV/r are second line drugs. Note:—* 0 value in the above table indicates patients who were on HAART naive not yet started drug treatment (DOCX) [file pone.0221853.s002.docx]

S2 Table

| ***Variables(n=411)*** | | ***Frequency*** | | ***Total*** |
| --- | --- | --- | --- | --- |
|  |  | ***On HAART (%)*** | ***On HAART naive (%)*** |  |
| ***Years lived with HIV*** | ***<1yr*** | ***13(4.2)*** | ***6 (5.8)*** | ***19(4.6)*** |
|  | ***1-5yrs*** | ***56(18.2)*** | ***84 (81.6)*** | ***140(34.1)*** |
|  | ***6-10yrs*** | ***154(50)*** | ***12 (11.7)*** | ***166(40.4)*** |
|  | ***>10yrs*** | ***85(27.6)*** | ***1 (1.0%)*** | ***86(20.9)*** |
| ***Morbidity identified*** | ***TB*** | ***3(1.0)*** | ***0*** | ***3(0.7)*** |
|  | ***Diarrheal diseases*** | ***6(1.9)*** | ***2(1.9)*** | ***8(1.9)*** |
|  | ***Candidiasis*** | ***5(1.62)*** | ***3(2.9)*** | ***8(1.9)*** |
|  | ***Malaria*** | ***1(0.3)*** | ***3(2.9)*** | ***4(1.0*** |
| ***CD4 cell count/ul*** | ***<200cells*** | ***43(14.0)*** | ***13(12.6)*** | ***56(13.6)*** |
|  | ***200-350 cells*** | ***80(26.0)*** | ***29(28.2)*** | ***109(26.5)*** |
|  | ***351-500*** | ***94(30.5)*** | ***23(22.3)*** | ***117(28.5*** |
|  | ***501+*** | ***91(29.5)*** | ***38(36.9)*** | ***129(31.4)*** |
| ***Duration of treatment in yrs.*** | ***5<yrs.*** | ***86(27.9%)*** | ***0**** | ***86(27.9%)*** |
|  | ***5-10 yrs.*** | ***152(49.4%)*** | ***0**** | ***152(49.4%)*** |
|  | ***>10 yrs.*** | ***70(22.7%)*** | ***0**** | ***70(22.7%)*** |
| ***Treatment type*** | ***TDF/3TC/EFV*** | ***165(53.6%)*** | ***0**** | ***165(53.6%)*** |
|  | ***TDF/3TC/NVP*** | ***41(13.3%)*** | ***0**** | ***41(13.3%)*** |
|  | ***AZT/3TC/NVP*** | ***44(14.2%)*** | ***0**** | ***44(14.2%)*** |
|  | ***AZT/3TC/EFV*** | ***34(11.0%)*** | ***0**** | ***34(11.0%)*** |
|  | ***TDF/3TC/Atazanvir/kalteza**** | ***20(6.5%)*** | ***0**** | ***20(6.5%)*** |
|  | ***AZT/3TC/Atzanavir/kalteza**** | ***2(0.6%)*** | ***0**** | ***2(0.6%)*** |
|  | ***ABC/3TC/ATV/r**** | ***2(0.6%)*** | ***0**** | ***2(0.6%)*** |
