# Supplementary material for: Anemia and its associated factors among adult people living with human immunodeficiency virus at Wolaita Sodo University teaching referral hospital
Source: PLoS One. 2019 Oct 9;14(10):e0221853. doi: 10.1371/journal.pone.0221853 (PMC6785157; doi:10.1371/journal.pone.0221853)
Supplement: S3 Table — BMI-Body Mass Index (DOCX) [file pone.0221853.s003.docx]

S3 Table

| ***Variable (n=411)*** | | ***Frequency*** | ***Percent (%)*** |
| --- | --- | --- | --- |
| ***BMI*** | ***<18.5*** | ***60*** | ***14.6*** |
|  | ***18.5-25*** | ***259*** | ***63.0*** |
|  | ***>25*** | ***92*** | ***22.4*** |
| ***Had eating difficult*** | ***Yes*** | ***17*** | ***4.1*** |
|  | ***No*** | ***394*** | ***95.9*** |
| ***Had nutritional counseling*** | ***Yes*** | ***388*** | ***94.4*** |
|  | ***No*** | ***23*** | ***5.6*** |
| ***Participated on nutritional support program*** | ***Yes*** | ***111*** | ***27.7*** |
|  | ***No*** | ***299*** | ***72.3*** |
| ***Frequency of eating /day*** | ***Twice/less*** | ***20*** | ***4.9*** |
|  | ***Three times*** | ***344*** | ***81.3*** |
|  | ***Four times or more*** | ***57*** | ***13.9*** |
| ***Mostly skipped meal*** | ***Not skipped*** | ***66*** | ***16.1*** |
|  | ***Breakfast*** | ***59*** | ***14.4*** |
|  | ***Lunch*** | ***32*** | ***7.8*** |
|  | ***Snack*** | ***252*** | ***61.3*** |
|  | ***Dinner*** | ***2*** | ***0.5*** |
| ***Dietary diversity*** | ***<3 food group*** | ***283*** | ***68.9*** |
|  | ***>3 food group*** | ***128*** | ***31.1*** |
