# Supplementary material for: Anemia and its associated factors among adult people living with human immunodeficiency virus at Wolaita Sodo University teaching referral hospital
Source: PLoS One. 2019 Oct 9;14(10):e0221853. doi: 10.1371/journal.pone.0221853 (PMC6785157; doi:10.1371/journal.pone.0221853)
Supplement: S4 Table — (DOCX) [file pone.0221853.s004.docx]

| ***Food item*** | ***Frequency of eating per week*** | ***Frequency*** | ***Total (%)*** |
| --- | --- | --- | --- |
| ***1. Cereal*** | ***>4/wk*** | ***386*** | ***93.9*** |
|  | ***<4/wk*** | ***25*** | ***6.1*** |
| ***2.Roots and tubers*** | ***>4/wk*** | ***281*** | ***68.4*** |
|  | ***<4/wk*** | ***130*** | ***31.6*** |
| ***3.Legume*** | ***>4/wk*** | ***250*** | ***60.8*** |
|  | ***<4/wk*** | ***161*** | ***39.2*** |
| ***4.Fruits*** | ***>4/wk*** | ***174*** | ***42.3*** |
|  | ***<4/wk*** | ***237*** | ***56.7*** |
| ***5.Meat*** | ***>4/wk*** | ***43*** | ***10.5*** |
|  | ***<4/wk*** | ***368*** | ***89.5*** |
| ***6.Vegetables*** | ***>4/wk*** | ***284*** | ***69.1*** |
|  | ***<4/wk*** | ***127*** | ***30.9*** |
| ***7.Milk and milk products*** | ***>4/wk*** | ***99*** | ***24.1*** |
|  | ***<4/wk*** | ***312*** | ***5.9*** |
| ***8.Egg*** | ***>4/wk*** | ***70*** | ***17.0*** |
|  | ***<4/wk*** | ***341*** | ***83.0*** |
| ***9.Fish*** | ***>1/month*** | ***44*** | ***10.7*** |
|  | ***<1month*** | ***367*** | ***89.3*** |

S4 Table
