# Supplementary material for: Anemia and its associated factors among adult people living with human immunodeficiency virus at Wolaita Sodo University teaching referral hospital
Source: PLoS One. 2019 Oct 9;14(10):e0221853. doi: 10.1371/journal.pone.0221853 (PMC6785157; doi:10.1371/journal.pone.0221853)
Supplement: S5 Table — (DOCX) [file pone.0221853.s005.docx]

S5 Table

| ***Classification***  ***anemia status*** | ***Male*** | | | ***Female*** | | | ***Overall***  ***( %)*** |
| --- | --- | --- | --- | --- | --- | --- | --- |
|  | ***HAART user (%)*** | ***HAART naïve (%)*** | ***Total***  ***%*** | ***HAART user (%)*** | ***HAART naïve (%)*** | ***Total*** |  |
| ***Non anemic*** | ***75(66.1)*** | ***22(53.7)*** | ***97(63.4)*** | ***129(65.8)*** | ***35(56.5)*** | ***164(63.4)*** | ***261(63.5)*** |
| ***Anemic*** | ***37(33.9)*** | ***19(46.3)*** | ***56(36.6)*** | ***67(34.2)*** | ***27(43.5)*** | ***94(36.4)*** | ***150(36.5)*** |
| ***1..Mild*** | ***28(75.7)*** | ***14(73.7)*** | ***42(75.0)*** | ***41(61.2)*** | ***23(85.0)*** | ***64(68.1)*** | ***106(70.7)*** |
| ***2. Moderate*** | ***9(8)*** | ***4(21.0)*** | ***13(23.2)*** | ***23(34.3)*** | ***4(14.8)*** | ***27(27.7)*** | ***40(26.7)*** |
| ***3.Severe anemia*** | ***0*** | ***1(5.3)*** | ***1(2.0)*** | ***3(4.5)*** | ***0*** | ***3(3.2)*** | ***4(2.6)*** |
