# Supplementary material for: Anemia and its associated factors among adult people living with human immunodeficiency virus at Wolaita Sodo University teaching referral hospital
Source: PLoS One. 2019 Oct 9;14(10):e0221853. doi: 10.1371/journal.pone.0221853 (PMC6785157; doi:10.1371/journal.pone.0221853)
Supplement: S6 Table — *P value ≤0.05 for AOR, BMI-Body Mass Index, HAART-Highly Active Antiretroviral Therapy, CD4-Cluster of Differentiation at T-cell. (DOCX) [file pone.0221853.s006.docx]

S6 Table

| ***Variables(N=308)*** | | ***Anemic*** | ***Non anemic*** | ***COR*** | ***P-value*** | ***AOR*** |
| --- | --- | --- | --- | --- | --- | --- |
| ***Sex*** | ***Male*** | ***46(30.9)*** | ***107(40.8)*** | ***0.65(0.42-0.99)*** | ***0.11*** | ***0.67(0.41-1.1)*** |
|  | ***Female*** | ***103(69.1)*** | ***155(59.2)*** | ***1*** |  | ***1*** |
| ***Marital status*** | ***Married*** | ***88(59.1)*** | ***186(71)*** | ***0.47(0.25-0.90)*** | ***0.18*** | ***0.55(0.23-1.32)*** |
|  | ***Single*** | ***22(14.8)*** | ***37(14.1)*** | ***0.59(0.27-1.31)*** | ***0.15*** | ***0.41(0.21-0.84)*** |
|  | ***Divorced*** | ***17(11.4)*** | ***17(6.5)*** | ***1(0.41-2.45)*** | ***0.5*** | ***0.73(0.27-1.92)*** |
|  | ***Widowed*** | ***22(14.8)*** | ***22(8.4)*** | ***1*** |  | ***1*** |
| ***Educational status*** | ***No Education*** | ***34(22.8*** | ***49(18.7)*** | ***0.72(0.37-1.41*** | ***0.14*** | ***0.57(0.26-1.22)*** |
|  | ***Primary Education*** | ***44(29.5)*** | ***86(32.8)*** | ***0.53(0.28-0.99)*** | ***0.29*** | ***0.47(0.23-0.93)*** |
|  | ***Secondary Education*** | ***42(28.2)*** | ***79(30)*** | ***0.45(0.24-0.84)*** | ***0.52*** | ***0.38(0.19-0.75)*** |
|  | ***Tertiary Education*** | ***29(19.5)*** | ***30(11.5)*** | ***1*** |  | ***1*** |
| ***HAART Status*** | ***HAART user*** | ***106(34.4)*** | ***202(65.6)*** | ***1*** |  | ***1*** |
|  | ***HAART Naive*** | ***43(41.7)*** | ***60(58.3)*** | ***1.37(0.87-2.16)*** | ***0.016**** | ***2.23(1.16-4.28)*** |
| ***Years lived with Virus*** | ***0-2 years*** | ***11(41.7)*** | ***32(12.2)*** | ***1*** |  | ***1*** |
|  | ***2-5years*** | ***42(28.2)*** | ***74(28.2)*** | ***1.65(0.76-3.6)*** | ***0.28*** | ***1.61(0.68-3.89)*** |
|  | ***5-8years*** | ***39(26.2)*** | ***62(23.7)*** | ***1.83(0.83-4.05)*** | ***0.05**** | ***2.59(1.02-6.57)*** |
|  | ***>=9 years*** | ***57(38.3)*** | ***94(35.9)*** | ***1.76(0.83-3.8)*** | ***0.04**** | ***2.6(1.03-6.59)*** |
| ***Frequency of eating*** | ***<3/day*** | ***135(90.6)*** | ***219(83.6)*** | ***1.89(1.00-3.59)*** | ***0.29*** | ***1.63(0.70-3.79)*** |
|  | ***>3/day*** | ***14(9.4)*** | ***43(16.4)*** | ***1*** |  | ***1*** |
| ***CD4 Count*** | ***<200*** | ***33(22.1)*** | ***23(8.8)*** | ***3.57(1.85-6.87)*** | ***<0.001**** | ***4.2(2.09-8.67)*** |
|  | ***200-350*** | ***44(29.5)*** | ***65(24.8)*** | ***1.68(0.98-2.89)*** | ***0.045**** | ***1.82(1.01-3.26)*** |
|  | ***351-500*** | ***35(23.5)*** | ***82(31.3)*** | ***1.06(0.61-1.84)*** | ***0.51*** | ***1.22(0.67-2.21)*** |
|  | ***501+*** | ***37(24.8)*** | ***92(35.1)*** | ***1*** |  | ***1*** |
| ***Infected with Intestinal parasite*** | ***Yes*** | ***25(16.8)*** | ***25(9.5)*** | ***1.91(1.05-3.45)*** | ***0.03**** | ***2.04(1.06-3.95)*** |
|  | ***No*** | ***124(83.2)*** | ***237(90.5)*** | ***1*** |  | ***1*** |
| ***BMI*** | ***<18.5*** | ***30(20.1)*** | ***30(11.5)*** | ***3.18(1.56-6.39)*** | ***0.006**** | ***2.96(1.37-6.39)*** |
|  | ***18.5-25*** | ***97(65.1)*** | ***162(61.8)*** | ***1.91(1.11-3.21)*** | ***0.02**** | ***1.98(1.11-3.56)*** |
|  | ***>25*** | ***22(14.8)*** | ***70(26.7)*** | ***1*** |  | ***1*** |
