# Supplementary material for: Anemia and its associated factors among adult people living with human immunodeficiency virus at Wolaita Sodo University teaching referral hospital
Source: PLoS One. 2019 Oct 9;14(10):e0221853. doi: 10.1371/journal.pone.0221853 (PMC6785157; doi:10.1371/journal.pone.0221853)
Supplement: S1 Study Tool — (DOCX) [file pone.0221853.s007.docx]

## Annex I

## Information Sheet

Wolaita Sodo University College of health science and MedicineSchool of public health

Name of the Principal Investigator: Temesgen Anjulo (Medical Laboratory Technologist, head of laboratory department in Wolaita Sodo University Teaching referral hospital.

This information sheet was prepared by Temesgen Anjulo investigator whose main aim is to study anemia and its associated factors among adult PLHIV attending ART clinic at Wolaita Sodo Referral Hospital.

Therefore, this research is to study the assessment Anemia and its associated factors among adult PLHIV. Hematological, immunological, parasitological and nutritional factors have their own impact for the occurrence and complication of anemia.The finding of this study will have their own benefit on treatment protocol and clinical efficacy of patients among PLHIV.

Procedure:Data was collected by structured questionnaires concerning the socio-demographic aspects questionnaires was asked. Clinical profiles were taken from the patient card.

For laboratory examination: 4ml of venous blood of blood samples were collected aseptically by one tube and CD4 enumeration conducted by FASC count machine and complete blood cell count will be assessed by automated hematological analyzer BS-3000 plus. There were no blood sample collected for this examination but all tests will be performed from patient’s routine follow up. Stool sample was collected and saline wet mount, at the same time Formal ether concentration technique was performed. All the clinical and laboratory examination results kept confidential using coding system whereby no one will have access to your clinical and laboratory results.

Risk and Discomfort: By participating in this research project, you may feel some discomfort when we request stool sample and we may take your time to ask some questions we will not take venous blood for this purpose from you but we will perform from your routine HIV care. Experienced health professionals will carry out all procedures.

Benefits: If you participate in this research, you may not get direct benefit but any treatable disease conditions diagnosed during the study period will be taken care of at no charge to you. In addition, your participation is likely to help us in understanding the study problem under investigation

Incentives: You will not be provided any incentives to take part in this research.

Confidentiality: The information that we collect from this research project will be kept confidential. Information about you that will be collected from the study will be stored in a file, which will not have your name on it, but a code number assigned to it. Which number belongs to which name will be kept under lock and key, and it will not be revealed to anyone except the principal investigator and if necessary your clinician.

Right to refuse or withdraw: You have full right to refuse from participating in this research if you do not wish to participate; and this will not affect your treatment or health services you get from this hospital in any way. You have also full right to withdraw from this participation at any time you wish to, without losing any of your rights as a patient in this Hospital.

Whom to contact? This research project will be reviewed and approved by WSU ethical review board. The main task of these institutional ethical committees is to make sure that research participants are protected from harm. If you want more information and check about this project, you can contactMr.Temsgen Anjulo (Masters of Public Health final year student) at Wolaita Sodo University (0949254387)

If you have, any questions PLEASE do not hesitate to contact and you may ask at any time you want.

## Annex II: Consent form

Code of study participant---------------------------------

Data collector name-------------------------

I have informed about a study that plans to investigate the prevalence of Anemia and associated factors among Adult PLHIV attending Wolaita Sodo Referral Hospital. Which will help in understanding the magnitude of anemia by assessing hematological, immunological, parasitological and other associated factors which will help to reduce the burden severity as well as improve the health status of the patient. For this study, I have been asked to give a gram of stool specimen and other investigations will be performed from blood sample from my routine care. I have informed that I will be asked some question related to the study. The investigator has briefed me that there are no major risks associated with the sampling procedure. I have also informed that there is no direct benefit provided to me. The investigator also informed me that all the laboratory results would be kept confidential. Moreover, I have informed of my right to withdraw from participating in this project and that my actions will have no impact on the overall management of my conditions. I have been given enough time to think over before I signed this informed consent. It is therefore, with full understanding of the situation that I gave my informed consent and cooperate at my will in the course of the conduct of the study.

Participant signature ------------------------------------------------

Data collector’s signature -----------------------------------------------

## Annex III Questionnaire

Code------------------------------ data collector------------------------------supervisor--------------------------

Part I. Socio-demographic status of study participants

| S.№ | Variables | Categorical codes | Frequency N (%) |
| --- | --- | --- | --- |
| 101 | Sex | Male-----1 |  |
|  |  | Female –2 |  |
| 102 | Age (in years) | _________ |  |
| 103 | Marital status | Single-------1 |  |
|  |  | Married -----2 |  |
|  |  | Divorced -----3 |  |
|  |  | Widowed ----4 |  |
|  |  | Other --------5 |  |
| 104 | What is your current residency? | Urban ---1 |  |
|  |  | Rural ---2 |  |
| 105 | What is your highest Educational level you attained? | No education ---0 |  |
|  |  | Primary --------1 |  |
|  |  | Secondary -----2 |  |
|  |  | Tertiary -------3 |  |
| 106 | What is your current Occupational? | 1. No occupation 2. employed 3. House wife 4. Merchant 5. Daily laborer   5.Other |  |
| 107 | Monthly income | --------------------------ETB |  |

PART-II. Patient clinical profile

| No | Variable | Categorical code | Skip |
| --- | --- | --- | --- |
| 201 | For how long have you been lived with HIV ? | --------------- |  |
| 202 | HAART status | 1. On HAART 2. Pre-ART |  |
| 203 | Duration of treatment in month | -------------- | From pt card |
| 204 | Baseline WHO stage | 1. I 2. II 3. III 4. IV | From card |
| 205 | Current WHO stage | 1. I 2. II 3. III 4. IV |  |
| 206 | Type of current drug regimen? | 1. TDF/3TC/EFV 2. TDF/3TC/NVP 3. AZT/3TC/NVP 4. AZT/3TC/EFV 5. d4T/3TC/NVP | From pt card |
| 207 | Had you Switched drug regimen? | 1. No 1.yes | If no skip to Q206 |
| 208 | Previous drug regimen?  Mostly taken regimen? | 1. TDF/3TC/EFV  2. TDF/3TC/NVP  3. AZT/3TC/NVP  4. AZT/3TC/EFV  5. d4T/3TC/NVP | Look card |
| 209 | Is there Current Co morbidity? | 1. No 1.yes | if no skip to 2011 |
| 210 | What is current co-morbidity patient suffering from? | 1. TB 2. Candidas 3. Malaria 4. Others | Look physical |
| 211 | Are you taking anti-TB drugs | 1. No 1. Yes | If no skip stop |
| 211 | Name of anti-TB drug | ------- | From pt card |

PART III. Anthropometric measurement and factors associated nutritional status

| No | Variable | Categorical code | Skip |
| --- | --- | --- | --- |
| 301 | Hieght (cm) | round 1 ----------round 2 ---------average------- |  |
| 302 | Weight(kg) | Round 1----------round 2-----------average------ |  |
| 303 | BMI | ------------ |  |
| 304 | Eating difficulty | 1. No 1. Yes |  |
| 305 | Dietary counseling | 1. No 1. Yes |  |
| 306 | Do you get nutritional support? | 1. No 1. Yes |  |
|  | Variable | Categorical code | Skip |
| 307 | Frequency of eating | 1. Twice/less /day 2. Three times /day 3. Four times or more/day |  |
| 308 | Breakfast | 1. Skipped 2. Not skipped |  |
| 309 | Lunch | 1. Skipped 2. Not skipped |  |
| 310 | Dinner | 1. Skipped 2. Not skipped |  |
| List all food items and drinks within last 24hrs | | |  |
|  |  | Raw material/ingredients |  |
|  | Breakfast |  |  |
|  |  |  |  |
|  |  |  |  |
|  |  |  |  |
|  | Lunch |  |  |
|  |  |  |  |
|  |  |  |  |
|  |  |  |  |
|  | Snack |  |  |
|  |  |  |  |
|  |  |  |  |
|  |  |  |  |
|  | Dinner |  |  |
|  |  |  |  |

Part IV.Measurement of hematological, immunological and parasitological findings

| sno | Variable | Range | Value | skip |
| --- | --- | --- | --- | --- |
| 401 | HGB (Baseline) | 13-18g/dl |  | From card |
| 402 | HGB(current) | 13-18g/dl |  |  |
| 403 | CD4 COUNT base line | 500-1300cells/ul blood |  | From card |
| 404 | Current CD4 Count | 500-1300cells/ul blood |  |  |
| 405 | Intestinal parasite | No o/p positive/specify parasites name | 1. Neg 2. Pos |  |
| 406 | Spp. of intestinal parasite ? |  | 1.E.histolytica  2.G.lambila  3.A.lumbrcoid  4.H.worm  5. S.stercolaris  6.others |  |
| 407 | Malaria | No hemoparasite or positive | 0.Neg 1.pos | If positive specific spp |
| 408 | If malaria is positive spp identified |  | 1.P.falciparium  2.P.vivax  3. other |  |
