# Supplementary material for: Anemia and its associated factors among adult people living with human immunodeficiency virus at Wolaita Sodo University teaching referral hospital
Source: PLoS One. 2019 Oct 9;14(10):e0221853. doi: 10.1371/journal.pone.0221853 (PMC6785157; doi:10.1371/journal.pone.0221853)
Supplement: S1 SPSS Out Put — (RTF) [file pone.0221853.s008.rtf]

Variables in the Equation	
		B	S.E.	Wald	df	Sig.	Exp(B)	95.0% C.I.for EXP(B)	
								Lower	Upper	
Step 1a	catyslvd3			5.040	3	.169				
	catyslvd3(1)	.475	.437	1.182	1	.277	1.608	.683	3.785	
	catyslvd3(2)	.950	.475	3.996	1	.046	2.587	1.019	6.569	
	catyslvd3(3)	.957	.474	4.072	1	.044	2.604	1.028	6.594	
	Sex(1)	-.397	.250	2.515	1	.113	.672	.412	1.098	
	Maritalstatus			7.105	3	.069				
	Maritalstatus(1)	-.600	.448	1.791	1	.181	.549	.228	1.322	
	Maritalstatus(2)	-.880	.360	5.969	1	.015	.415	.205	.840	
	Maritalstatus(3)	-.321	.496	.419	1	.517	.725	.274	1.917	
	Educationallevel			8.273	3	.041				
	Educationallevel(1)	-.571	.391	2.134	1	.144	.565	.263	1.215	
	Educationallevel(2)	-.767	.351	4.766	1	.029	.465	.233	.925	
	Educationallevel(3)	-.979	.350	7.808	1	.005	.376	.189	.747	
	HAARTstatus(1)	.803	.332	5.846	1	.016	2.233	1.164	4.283	
	Intestinalparasite1(1)	.715	.336	4.532	1	.033	2.044	1.058	3.947	
	cd4current			16.687	3	.001				
	cd4current(1)	1.434	.371	14.966	1	.000	4.194	2.029	8.673	
	cd4current(2)	.598	.298	4.021	1	.045	1.818	1.014	3.259	
	cd4current(3)	.198	.303	.427	1	.513	1.219	.673	2.206	
	BMIgrouped			8.251	2	.016				
	BMIgrouped(1)	1.085	.392	7.648	1	.006	2.961	1.372	6.390	
	BMIgrouped(2)	.684	.298	5.275	1	.022	1.982	1.106	3.555	
	Constant	-1.120	.671	2.782	1	.095	.326			
a. Variable(s) entered on step 1: catyslvd3, Sex, Maritalstatus, Educationallevel, HAARTstatus, Intestinalparasite1, cd4current, BMIgrouped.	

Cat year's lived- reference-fist                      CDA current- indicator
HAART status-reference-fist                        Marital staus-indicator 
Se-indicator
Intestinal parasite-references fist
Education –indicator
BMI- indicator
