# Supplementary material for: Anemia and its associated factors among adult people living with human immunodeficiency virus at Wolaita Sodo University teaching referral hospital
Source: PLoS One. 2019 Oct 9;14(10):e0221853. doi: 10.1371/journal.pone.0221853 (PMC6785157; doi:10.1371/journal.pone.0221853)
Supplement: S1 SPSS Descriptives Out Put — (DOC) [file pone.0221853.s009.doc]

                  Descriptive out put 
Sex	
		Frequency	Percent	Valid Percent	Cumulative Percent	
Valid	1	153	37.2	37.2	37.2	
	2	258	62.8	62.8	100.0	
	Total	411	100.0	100.0		


Marital status	
		Frequency	Percent	Valid Percent	Cumulative Percent	
Valid	1	59	14.4	14.4	14.4	
	2	274	66.7	66.7	81.0	
	3	34	8.3	8.3	89.3	
	4	44	10.7	10.7	100.0	
	Total	411	100.0	100.0		


Educationallevel	
		Frequency	Percent	Valid Percent	Cumulative Percent	
Valid	0	83	20.2	20.2	20.2	
	1	130	31.6	31.6	51.8	
	2	139	33.8	33.8	85.6	
	3	59	14.4	14.4	100.0	
	Total	411	100.0	100.0		


Residency	
		Frequency	Percent	Valid Percent	Cumulative Percent	
Valid	1	365	88.8	88.8	88.8	
	2	46	11.2	11.2	100.0	
	Total	411	100.0	100.0		


cd4current	
		Frequency	Percent	Valid Percent	Cumulative Percent	
Valid	<200	56	13.6	13.6	13.6	
	200-350	109	26.5	26.5	40.1	
	351-500	117	28.5	28.5	68.6	
	501+	129	31.4	31.4	100.0	
	Total	411	100.0	100.0		


Age group	
		Frequency	Percent	Valid Percent	Cumulative Percent	
Valid	15-24	39	9.5	9.5	9.5	
	25-34	155	37.7	37.7	47.2	
	35-44	153	37.2	37.2	84.4	
	45-54	53	12.9	12.9	97.3	
	55+	11	2.7	2.7	100.0	
	Total	411	100.0	100.0		


Monthly income	
		Frequency	Percent	Valid Percent	Cumulative Percent	
Valid	<750	256	62.3	62.3	62.3	
	750-1500	55	13.4	13.4	75.7	
	1501-3000	66	16.1	16.1	91.7	
	3001-4500	14	3.4	3.4	95.1	
	4501+	20	4.9	4.9	100.0	
	Total	411	100.0	100.0		


HAART status	
		Frequency	Percent	Valid Percent	Cumulative Percent	
Valid	1	308	74.9	74.9	74.9	
	2	103	25.1	25.1	100.0	
	Total	411	100.0	100.0		


Intestinalparasite1	
		Frequency	Percent	Valid Percent	Cumulative Percent	
Valid	0	361	87.8	87.8	87.8	
	1	50	12.2	12.2	100.0	
	Total	411	100.0	100.0		
